# 

**Published:** 1899-12

**Authors:** 


### CONTENTS.

ORIGINAL COMMUNICATIONS—

THE BUILDING OF ATLANTA. By Bernard Wolff, M.D., Atlanta, Ga. .... 649
RELATION OF BOARD OF HEALTH TO THE CITY AND MEDICAL PROFESSION. By R. R. Kime, M.D., Atlanta, Ga. .... 651
THE PHYSIOLOGICAL THERAPEUTICS OF HEART-FAILURE. By C. A. F. Lindorm, Ph.D., M.D., Chicago, Ill. 656
CONDITIONS SUCCESSFULLY TREATED BY ELECTROLYSIS. By M. B. Hutchins, M.D., Atlanta, Ga. .... 662
THE DIFFERENT PHASES OF ELECTRIC TREATMENT. By J. McFadden Gaston, M.D., Atlanta, Ga. .... 667

SOCIETY REPORTS—

THE CLINICAL SOCIETY OF MARYLAND .... 676

EDITORIAL NOTES AND COMMENTS—

QUACKERY IN GEORGIA .... 685
STATE BOARD OF HEALTH .... 688

NEWS AND NOTES .... 694
CONTENTS CONTINUED .... x

